# From P4 medicine to P5 medicine: transitional times for a more human-centric approach to AI-based tools for hospitals of tomorrow

**DOI:** 10.12688/openreseurope.14524.1

**Published:** 2022-03-09

**Authors:** Denise Amram, Arianna Cignoni, Tommaso Banfi, Gastone Ciuti

**Affiliations:** 1 LIDER LAB - DIRPOLIS Institute EMbeDS Department of Excellence, Scuola Superiore Sant'Anna di Studi Universitari e di Perfezionamento, Pisa, Pisa, 56100, Italy; 2 The BioRobotics Institute Department of Excellence in Robotics & AI,, Scuola Superiore Sant'Anna di Studi Universitari e di Perfezionamento, Pisa, Pisa, 56100, Italy

**Keywords:** Artificial Intelligence (AI) – Assessment – ALTAI checklist – Healthcare sector

## Abstract

Within the debate on shaping future clinical services, where different robotics and artificial intelligence (AI) based technologies are integrated to perform tasks, the authors take the chance to provide an interdisciplinary analysis required to validate a tool aiming at supporting the melanoma cancer diagnosis. In particular, they focus on the ethical-legal and technical requirements needed to address the Assessment List on Trustworthy AI (ALTAI), highlighting some pros and cons of the adopted self-assessment checklist. The dialogue stimulates additionally remarks on the EU regulatory initiatives on AI in the healthcare systems.

## Disclaimer

The views expressed in this article are those of the author(s). Publication in Open Research Europe does not imply endorsement of the European Commission.

## Plain language summary

The development of AI-based technologies for healthcare services represents a new frontier of the current innovation process. In order to ensure the highest protection and enhancement of individual and collective fundamental rights, ethical-legal requirements shall be addressed by design.

## Introduction

In the last year, the EU accelerated the legislative process on artificial intelligence (AI).

In July 2020, the High-Level Expert Group on Artificial Intelligence (AI HLEG) presented the final Assessment List for Trustworthy Artificial Intelligence (ALTAI) ^1^. While, in October 2020, the European Parliament adopted a “Provisional text on the *Framework of ethical aspects of artificial intelligence, robotics and related technologies*”, where the risk-based approach is confirmed as main strategy for the further AI legislative initiatives that the EU Commission is working on (hereinafter “Provisional Resolution”) ^2^. On April 21 ^st^ 2021 both a Proposal for AI Regulation and a Proposal for Machinery Products Regulation were published ^3^. At the end of November 2021, the Presidency of the Council of the European Union published some amendments to be included in the current Proposal of AI Regulation.

The common core of these initiatives refers to the adoption of a risk-based approach towards fundamental rights protection. In particular, to assess risks and implement corresponding mitigation actions will help to identify specific roles and responsibilities in each step of the design of a given technology. This is true especially in the healthcare sector, where innovative technologies are impacting on vulnerable subjects. From this perspective, a deep analysis of the ALTAI methodology and structure becomes crucial to address in a responsible and proactive way the current compliance challenges for those who develop AI-based systems to shape the future healthcare facilities and services ^4^.

ALTAI consists of a series of questions that may steer ^5^ the AI designers ( *rectius* the AI-controller, or developers according to the mentioned Provisional Resolution) towards a multidisciplinary evaluation path aimed at addressing the seven ethical-legal-safety compliance challenges emerging by the Guidelines on Trustworthy AI, adopted by the EU Commission in April 2019 ^6^, and considered as the state-of-the-art of such an assessment in the AI Proposal of Regulation.

According to them, an AI system becomes trustworthy when it is lawful ( *i.e.*, compliant with the applicable legal framework), ethical ( *i.e.*, compliant with the applicable ethical framework) and robust ( *i.e.*, compliant with the applicable safety standards). The interplay between these three pillars ^7^ is determined by the following seven grounds ^8^.

1. *Human Agency and Oversight*: it includes both the ethical and the legal dimension as it refers to fundamental rights protection aimed at maintaining the balance between human control and technical progress in terms of human agency and oversight. Human beings shall be protected both as individuals and groups, taking into account inclusiveness, fairness, non-discrimination and vulnerabilities protection as paramount interests.
2. *Technical Robustness and Safety*: it refers to the system resilience to attacks and security, including fall back plans and the compliance with the highest levels of general safety, accuracy, reliability, and reproducibility.
3. *Privacy and Data Governance*: this profile establishes a bridge with the most effective compliance process by design and by default introduced for personal data processing by the EU Reg. n. 2016/679 on General Data Protection Regulation (GDPR) aiming at guaranteeing the respect for confidentiality, quality, and integrity of data.
4. *Transparency*: this is a principle established to guarantee the traceability, explainability, communication of methods, goals, and results of the given AI system.
5. *Diversity, Non-discrimination, and Fairness*: this ground refers to the interdisciplinary safeguards to be implemented in order to avoid a misuse or an unfair use of AI in terms of bias, accessibility, and universal design.
6. *Societal and Environmental Well-being*: the AI system shall be put in the market as a sustainable solution under the environmental, social, and societal perspectives, considering the democratic values rooted within the EU framework.
7. *Accountability*: this is the main principle that enables the compliance process in terms of proactively responsibilities allocation through a risk-based approach that includes auditability, minimization and reporting of negative impact, trade-offs, and redress.

In order to undertake the ALTAI checklist, a web-based tool that provides results in terms of level of compliance and list of recommendations has been developed. This could be followed by anyone who is responsible to design and develop an AI system to confidentially perform a self-assessment. This preparatory tool can be interpreted as a pilot for possible future obligations on the topic, as the risk-based approach addressed through an impact assessment seems to be suggested by the above-mentioned EU Parliament Resolution as well.

In the following paragraphs, we will assess the ALTAI checklist on possible design of AI-tools applied to healthcare, considering the paramount role that the health-data debate has among the European strategy for data ^9^ and the related investments to encourage the upscaling of cross-border exchange of health data and their re-use to improve the detection, diagnosis, and treatment of diseases.

In particular, we will provide some methodological remarks emerging from the application of the ALTAI to an AI-based tool developed through a Predictive, Preventive, Personalised, and Participatory (P4) cancer medicine approach, in order to contribute to the debate from a bottom up and interdisciplinary perspective. In fact, within this empirical assessment, our efforts focused on both explaining the tool design from a scientific viewpoint, covering engineering, ethical-legal, and medical aspects and on introducing technical and organizational enablers to allow the real participation of unexpert users in the prevention of specific pathologies.

According to the main digitalization challenge of healthcare, we addressed our remarks to boost outcomes personalization features – based on the user/patient ones – starting from the contribution that high level datasets of users’/patients’ health-data could bring to the enhancement of the healthcare sector. Sensitive health-data processing, indeed, plays a crucial role in the development and deployment of these specific tools, that impact both on patients as a vulnerable group, and on individuals. In the event that the AI-based technologies would support wrong evaluations or would provide non-compliant data management strategies, they could lead to misjudgements and adverse outcomes. Consequences might be envisaged not only in terms of physical harm for a given patient, but they could also affect the psychological dimension of a given patient/end-user. For this reason, our remarks deal with a further challenge, as hereinafter we will refer to the so-called P5 medicine ^10^, including additionally “Psycho-cognitive” aspects arising from the commented technologies to the Predictive, Preventive, Personalised, and Participatory ones addressed in P4 medicine.

In this paper, therefore, we will deal with common issues – ranging from ethical-legal to technical ones – to properly address the compliance activities related to the technical, as well as ethical-legal, challenges emerging in the context of AI-based tools development for the implementation of an effective P5 medicine. At the same time, we will suggest possible solutions to cover gaps emerging from the available ethical-legal assessment tools.

## Preliminary activities to perform a responsible ALTAI.

ALTAI is a method to drive the self-assessment of the trustworthiness of a given AI-based technology and it is based on 63 questions divided into the above-illustrated seven key requirements. Interdisciplinary expertise is required to answer all questions. A closed yes/no answer is not sufficient, in fact, to perform the analysis and achieve a conscious and resolute opinion about the level of trustworthiness of the designed tool ^11^.

For example, the first block of questions (Q1–Q6.2) is focused on assessing the impact of the designed application on fundamental rights. Therefore, a deep knowledge of what is meant by fundamental rights protection and how to assess the corresponding impact shall be introduced in the evaluation workflow ^12^. Furthermore, to properly answer the second block (Q7–Q18.5), a deep knowledge of cybersecurity and safety standards is required as, again, yes/no answers are not sufficient to the whole trustworthy evaluation process. In particular, the AI-designer shall justify the reasons that caused them to choose a given technical measure as well as to implement a specific safeguard over a different one under several grounds, including human safety, animal protection, environment, security, and misuse. Within the same context, the designer is required to identify a “fallback plan” aimed at ensuring the maintenance of an acceptable level of risk if something goes wrong. Those evaluations are functional also in a prognostic perspective to answer questions included in the sixth block (Q49–Q53), related to societal and environmental wellbeing ^13^. Therefore, the trustworthiness combines the need to prevent harms as well as the need to address the current multi-faced challenges of societal empowerment ^14^.

The third block (Q19–Q29.3) recalls the GDPR compliance ^15^: the results of the data protection assessment performed under article 35 GDPR for the given personal data processing shall be confirmed within the more comprehensive framework of the AI-based system. In this context, the data protection officer’s (DPO) involvement is not only suggested, but also an organizational measure to be assessed in order to reach the trustworthy standard of the developed ecosystem. As a preliminary activity, the AI designer/developer has to verify whether or not a data protection officer and/or a privacy expert shall be consulted and appointed. This profile is strongly connected with the first block as the impact on the other fundamental rights reasonably stands in a cause-effect relationship with the protection of the confidentiality, availability, and integrity of personal data. For instance, a data breach concerning an AI-application for patients could also infringe on their health, private life, dignity, etc., but it could also identify possible grounds of discrimination according to the end users’ vulnerability. This last profile on fairness is addressed by the fifth block (Q41–Q48) of the check-list assessment.

The fourth block (Q30–Q40.2) on transparency consists of a series of questions related to the development of each AI-based system, considering it as the result of a series of human decisions taken by the AI controller. In a binding legal framework that defines roles and responsibilities, he/she may assume the role of AI-controller, similarly to the data controller as defined by article 5 GDPR ^16^. AI-based systems, in fact, firstly include the identification of methods for data acquisition regarding the function/algorithm that must be applied to a previously determined dataset. Secondly, the AI-controller shall define what tasks shall the AI perform (the so-called *required actions*) as well as the final purposes ( *goals*) of the automated decision making/reasoning activities.

The last block (Q54–Q63) will assess the overall level of accountability of the process, suggesting the implementation of organizational measures aimed at monitoring the process and also providing solutions in case of issues.

A first step to be performed by the AI controller/developer is to engage an interdisciplinary team aimed at strengthening a dialogue and share best practices for the ALTAI purposes. Furthermore, an independent advisor might support the assessment in order to interpret notions and corresponding adjectives ( *e.g.*, what is meant for “meaningful interactions and appropriate human oversight and control” in Question n. 4, “an adequate working definition of fairness” in Question n. 44, “wide range of individual preferences” in Question n. 45).

Secondly, the identified specific parameters and standards shall drive the overall assessment and provide a robust and coherent internal framework of reference. This is true also for non-technical requirements, whose harmonized application shall emerge from the individual answers given to each block. For instance, if we identify a ground of vulnerability in respect to the impact on fundamental rights, the same analysis shall be reproduced within the assessment of fairness as well as within the governance-related issues and social impact ones ^17^.

Once the technical and organizational measures to reach the acceptable level of trustworthiness of the AI-ecosystem have been implemented, a continuous evaluation system shall be maintained in order to ensure upgrades both in terms of performance and enhancement of fundamental rights. To this end, proper mechanisms of check and balance could be introduced within codes of conduct, encouraged by article 40 GDPR for personal data processing. They currently provide a compliance support for small and medium enterprises, addressing common issues and challenges in terms of self-regulation and best practices not only for personal data processing, but for the development of AI-based systems as well ^18^.

These preliminary remarks shall orient the AI-controller/developer towards a legally attentive design of the given application.

In the following paragraphs, we will present the results of a discussion between an interdisciplinary group, including scholars in law, biomedical engineering, and computer science, on how to develop a trustworthy AI-based tool aimed at early detection of melanoma skin cancer.

The digitalization of the healthcare services, in fact, is boosted where existing data flows can be re-usable to train (such as in the case study we will address below) an algorithm that could process information in order to predict a decision. These tools shall be enabled within a robust data governance ecosystem aimed at providing the predictive healthcare service and, at the same time, ensuring the exercise of end-users’ rights. Data collection, processing, and storage shall, therefore, allow mechanisms of re-training of the algorithms, while providing a specific prediction/decision making result for the user/users. The interoperability for data formats and the enhancement of data security shall be combined with acceptable levels of pseudonymisation and anonymisation for the training, as well as on the linking and de-linking of records for the given query.

These remarks become crucial to enable innovative solutions and care delivery, including the opportunity for patients to control and administer care themselves. Considering that the P5 medicine includes keywords such as participative, preventive and personalised, highlighting how the participation of the patient is paramount to preventing adverse outcomes in pathologies’ development, our aim is to assess the ALTAI while assessing a given technology in a mutual exchange of technical and organizational good practices. Under the purpose to overcome possible practical issues emerging from this innovative mechanism of assessment and, at the same time, to properly address the ethical-legal compliance in R&D&I (Research, Development, and Innovation) sectors, we will highlight weaknesses and strengthens of the proposed structured evaluation system ^19^.

## Building up a trustworthy AI-based tool for P5 medicine.

Our analysis starts from the need to develop an ethical-legal by design and by default AI-based tool to support the early-detection of melanomas.

Melanoma has the highest mortality rate among skin cancers ^20^, and it can grow from the early stage (called melanoma *in situ*) to the latest stage (metastatic melanoma) in a period ranging from 8 to 12 months ^21^. An early diagnosis is essential to improve the survival rate ^22^ and reduce treatments costs ^23^. Since this pathology appears on the skin surface, it can be detected by monitoring changes of the skin itself. This condition has peculiar morphological attributes, and an expert clinician is needed to make a diagnosis. Nevertheless, melanoma lesions are not easy to detect in its early stage when these lesions present borderline features, even to an expert eye ^24^. AI technology may aid clinicians in the melanoma diagnosis, especially in its earlier stage. The use of the smartphone, that has become a large-scale deployable tool, paired with this kind of technology may enable a common user to take an active and participative role in skin cancer prevention.

As anticipated, despite the internal allocation of technical tasks, the AI-controller/developer shall identify a series of roles aimed at giving advice and being responsible for some specific tasks within the ALTAI context.

The first preliminary issue is the allocation of human resources in the design process. From a law and policy making perspective, indeed, incentive mechanisms need to be identified in order to overcome the lack of effectiveness of a (still) unbinding approach. In this regard, the GDPR legislative model seems particularly effective, as it includes a series of binding obligations framed within a structured illustration of principles, roles, and enforcing tools. In addition, a frame of optional mechanisms and safeguards that each legal system may decide to regulate or not.

The second transversal issue is related to establishment of a multi-level governance system. This is functional to identify a monitoring process and coordinate the engagement of the previously identified roles.

In the analysed P5 medicine tool, a technical board that includes medical doctors, biomedical engineers, software engineers, data protection and ethics experts shall be established to interpret the different interdisciplinary key requirements that the ALTAI assessment provides. This technical board shall be able to raise issues and identify solutions in light of a general mutual purpose to co-create a new technology enhancing fundamental rights protection. Such a technical board could also host different experts when seeking external advice as well as including stakeholders and end-users’ opinions to assess different solutions.

Furthermore, the multilevel governance shall comply with the applicable legal framework that means for example that the GDPR governance in terms of appointment of joint controllers and data processors shall be structured according to the security governance determined by the specific standards followed by the developer. In addition, further engagements are envisaged by each block of questions. This may contribute to shared responsibilities and to prove the overall accountability within the process.

The framework becomes more complex in the case of public/private stakeholders as well as in the case of cross-border relationships between the identified players since the national compliance process may present some gaps/overlapping profiles. In this regard, the possible legislative misalignment shall be addressed and covered during the assessment by specific agreements between the involved parties.

The third step is end-users centred. In fact, compliance activities shall deal with the main features that characterise those persons or groups of persons whose data are processed ( *i.e.*, the data subjects under the GDPR) and those who are the addressees of the prediction/automated decision-making process. The two categories might sometimes overlap, but they usually do not. The ALTAI process shall deal with all possible end-users both to protect and enhance their rights. To this end, the technical board shall be open to collect feedback as well to include a validation step with end-users to collect feedback not only on the technical level related to the automated decision-making, but also on its usability. In fact, in our example an AI-based tool will be addressed both to clinicians and patients with evident differences in terms of awareness, risks, benefits, and impact on corresponding rights. For instance, health protection will be assessed in terms of individual fundamental rights for each patient and in the collective dimension as far as the clinicians are concerned. At the same time, the supportive role of the AI shall be properly addressed in order to do not make the human decision too overconfident or, on the contrary, causing replacement distress among professionals.

Once these profiles have been addressed as priorities by the AI-controller/developer, the ALTAI might be filled.

## ALTAI gaps and strengths

Questions developed within the ALTAI checklist have been interpreted to design an AI-based tool aimed at detecting melanoma early by a decision-making system that processes images, in order to highlight gaps and strengths of the checklist.

Table 1 below shows the results of the ALTAI analysis and possible comments that may either address good practices or issues

**Table 1.** ALTAI requirements and comments. (The first column identifies the requirements according to the ALTAI checklist, the second column provides details on how each requirement is met under the example, the third column adds some comments by the authors to better highlight the ethics, lawfulness, robustness aspects arising from the assessment. Legend. AI: artificial intelligence; GDPR: general data protection regulation; DPOs: data protection officers; GPUs: graphics processing units; P5: Predictive, Preventive, Personalised, and Participatory and Psycho-cognitive, R&D&I: research, development, and innovation).

Human agency and oversight	Answer	Comments
*Fundamental rights*	Patients’ fundamental rights involved are dignity, health, data protection. Clinicians’ fundamental rights involved are dignity and work-life. The decision-making process could interact with patients, stimulating their awareness towards the risk of melanoma, suggesting contacting a clinician. The tool could recommend the patient get clinical advice and therefore it could interfere with the patient's decision to get or not to get clinical advice. Individual vulnerabilities shall be addressed in the information section before using the tool. The clinicians may be supported in the pre-screening activities, but diagnosis shall be performed under the current clinical practice.	This key requirement arises from the need to identify the list of fundamental rights. The list could be included within the Terms & Conditions of the given system/device/tool to accomplish an extensive information duty. A possible survey could be implemented to identify whether or not the end-user is vulnerable as well as her/his attitudes towards the results of the decision-making system ( *e.g.*, if the end-user declares they use drugs or are in a temporarily vulnerable state for some reason and the use of the tool may cause undesirable consequences, it is important to avoid any discomfort caused by the tool). Communication interfaces shall be addressed in a clear and user- friendly format.
2 *Human agency*	The AI system shall support the cancer prevention actions of the healthcare system, revising internal processes in light of such support in the pre-screening activities. No risks of overreliance and overconfidence in the AI system, as it gives just pre-screening information. Clinical diagnosis shall in any case be provided through gold standard methodologies.	Disclaimer on the system characteristics shall be included both in the Terms & Conditions and in the Handbook. Awareness and training campaigns shall be promoted among clinicians.
3 *Human oversight*	Human control is granted as the AI system intervenes on a pre-screening step, as it could only recommend a dermatologic consult in the case that it is required by a given outcome.	A possible follow-up survey could be delivered to clinicians and patients to provide further validation on the test.
**Technical robustness** **and safety ^25^**	**Answer**	**Comments**
*Resilience to attack and* *security*	A monolithic system ensures a safer ecosystem. Regarding AI models, specific technical methodologies ^26^ may be implemented to ameliorate model robustness against external perturbations and attacks. A further layer of security might be implemented in the model itself by (1) using *ad hoc* methods enabling the use on encrypted data as input to certain models, and (2) adopting models and libraries optimized for privacy sensitive applications ( *e.g.*, Opacus).	Stress tests shall be scheduled to assess the overall resilience of the system to attacks and breaches.
*Fallback plan and* *general safety*	A fallback plan aligned to the general backup policy is applied. As the learnability of a specific problem is uncertain a-priori ^27^, a rigorous scientific methodology should be applied to the evaluation of a solution performance. To gauge the level of uncertainty associated with a specific prediction of a specific model, and hence to mitigate the effect of identifiable unreliable predictions, specific methodological approaches may be used ( *e.g.* as reported in previous studies ^28– 30^).	Adding redundancy in the software and hardware components of the system may also be a viable and widely adopted strategy to boost the overall system safety and ensure prompt recovery from anomalies, also enabling internal and automatic system diagnostic *e.g.* as done in avionics systems or, regarding AI models, using an ensemble of models.
*Accuracy*	In general, a specific AI algorithm architecture is used to solve a certain task and needs a specific metric to correctly evaluate its performances. The AI-controller/ developer is responsible for identifing the proper statistical method for the specific case of implementation. For instance, a single shot detector model ^29^ is used for object detection problems and its performances are evaluated using mean average precision metric. As an example, in the specific case of classification task, the tool accuracy is evaluated as the ratio between the sum of True Positives and True Negatives values scored by the algorithm on the training data and the total number of training samples. Other metrics, such as sensitivity (ratio between True Positives and all Positive samples) and specificity (ratio between True Negative and all Negative samples), are computed to better understand the learning level of the implemented algorithms.	In the current application, true Positives represent the number of positive samples ( *i.e.,* patients with melanoma) that are scored by the tool correctly affected by the pathology. True Negatives represent the number of negative samples ( *i.e.,* patients not affected by melanoma) the tool scores as healthy. In this field of application ( *i.e.,* melanoma detection), sensitivity is usually maximized at the expense of specificity.
*Reliability and* *reproducibility*	A specific monitoring system has been implemented to assess the algorithm performance. Code shall be extensively documented and it could be versioned using an open-source implementation for version management based on Git (website: https://git-scm.com/).	Extensive and detailed documentation of the scientific methodology followed to build and validate a specific AI solution should always be produced to ensure that independent reproduction and scrutiny of the technical implementation is feasible ^31^. When applicable, the source code of the specific implementation should also be made available at least for external technical review ^32^.
**Privacy and data** **governance**	**Answer**	**Comments**
*Respect for privacy and* *data protection*	End-users' consent is the legal basis of the data processing provided with the tool. As a consequence, technical and organizational measures shall be implemented to inform, and to let the data subject exercise her/his rights. For instance, the interface will allow to enable/disable both for algorithm continuous training and the decision-making process anytime. A data protection impact assessment is performed and risks for the availability, confidentiality, integrity of data shall be mitigated in terms of acceptability. The DPOs shall be involved in the process. Within the step of training of the AI-tool: data governance and ownership shall be governed in compliance with the applicable legal framework. Therefore, data shall be anonymous or, in case of pseudonymised ones in the frame of a collection provided directly from trials in the clinics, flows shall be encrypted, pseudonymisation techniques shall be applied and the involvement of the competent ethics committees shall be included in the process.	Within this section, the AI-assessment deals with all the GDPR compliance activities. These activities include the identification of the data governance, the security measures, and the identification of roles and responsibilities that ensure that all data processes are provided in light of the principles of lawfulness, fairness, transparency, accountability, purposes limitation, data minimisation, and accuracy. For those AI-controllers/developers that are not processing personal data, they shall be compliant with the EU Regulation (EU) 2018/1807 of the European Parliament and of the Council of 14 November 2018 on a framework for the free flow of non-personal data in the European Union, that entered into force on 28 May 2019 ^33^.
*Quality and integrity of* *data*	The development of the tool is framed in a detailed privacy governance policy, following a tailored management/authorization path.	Data pooling activities shall pay attention to the possibility that to process information by crossing anonymous and pseudonymised datasets may incur the risk of re-identification or, at least, the necessity to regulate data flows through specific data sharing agreements.
*Access to data*	Dedicated servers, with limited access, host the development of the tool and anonymised/pseudonymised data.	The internal and external governance as well as technical measures shall be identified, implemented and maintained for the entire life cycle of the data according to the results of the impact assessment.
**Transparency ^34^**	**Answer**	**Comments**
*Traceability*	User is guided in the data gathering process ( *e.g.,* using viewfinder to centre the skin lesion, checking the blur of the image), ensuring the correct data is gathered. This is essential to guarantee the correct pre-processing phase, before feeding the algorithm using the user’s data. Decision-making outputs can be monitored using thresholds. As an example, in case of probability as output, data that gives as output probabilities within certain ranges, raising concerns on the reliability of the algorithm output, are forwarded to a clinician, as described in the Communication requirement. These data paired with the clinician response ( *i.e.,* the ground truth or target) enlarge the original dataset that it is used to re-train the AI algorithm. Here, metrics described in the Accuracy section are used to evaluate the algorithm training, monitoring its performances.	Traceability is fundamental to address the transparency of the AI- system in order to verify the quality of the decision-making outputs. AI algorithms are strongly dependent on the data format and quality used to train them to boost their learning ability and produce the desired result (in this case, detecting a malignant skin lesion). The format and quality must be similar to the ones the algorithm uses during the training phase. AI algorithms can be considered “black boxes”, since they process data in ways that are not easily audited or understood by human. This limits the traceability of what happens within the algorithm. Nevertheless, strategies can be implemented to monitor the data quality and the algorithm performances (e.g. blockchain technologies) ^35^.
*Explainability ^36^*	A viable inclusion of computer-aided design system in melanoma diagnosis pipeline was shown by Waterhouse *et al*. in 2019 ^37^. The AI-decision may influence the screening activities, according to the user's experience, including clinicians. As an alternative, other recent methods tend to “open” black boxes models such as AI algorithms. Here ^38^ the authors provided a classification of approaches to interpret the decision-making process based on the problem definition and the black box type.	The experience plays a crucial role in trusting an AI-based decision. Non-expert users tend to follow the results output by the AI tool. This is also true for clinicians. Indeed, it has been shown that clinicians with the least experience follow the AI tool’s decisions if it contradicted their initial diagnosis, even if they were confident. However, faulty AI can mislead both experienced and unexperienced clinicians. This is an aspect to consider when deploying these tools. This could be overcome providing additional features to the clinician ( *e.g.*, for the current application: asymmetry index, border irregularity index) that can aid their final decision.
*Communication*	The interface shall be developed in accordance with the given task and the involved roles (clinicians or end users).	It has been demonstrated that for clinicians it is better to show multiclass probability when dealing with multiclass diagnosis. Conversely, malignancy probability can be used to manage binary decisions ( *e.g.,* whether or not do a biopsy, whether or not go visit an expert clinician) ^39^. As described in the “explainability” requirement, non-expert users tend to follow the AI results, even in the case of a wrong suggestion. Melanoma growth happens within months, thus missing the detection of this lesion type can lead to adverse outcomes. The solely probability of the decision-making result included in the answer provided could be ambiguous information to be interpreted by a clinician, which is true especially in case of intermediate values. In an effort to maximize the sensitivity of the tool, giving clear responses to the user, the clinician himself/herself can be involved in this detection process ^40^. In case of probability/ uncertainty, the lesion data could be forwarded to a clinician for an additional evaluation. The clinician’s decision is then used as the final response to the user as well as to improve the AI algorithm performances. Indeed, the new data, paired with the clinician response ( *i.e.,* the ground truth or target), enlarges the original training dataset and it is used to re-train the AI algorithm.
**Diversity and non-** **discrimination**	**Answer**	**Comments**
*Unfair bias avoidance*	Biases are possible. Biases are mainly related to the personal characteristics that compose the training datasets. Melanoma incidence rate is higher in people with lower phototypes ( *e.g.,* white populations), while is almost rare in those with higher ones ( *e.g.,* black populations). Datasets will thus be biased on skin colour. Data gathering and assumptions made when training an algorithm can also introduce biases, thereby distorting the final output. These are important aspects to consider when dealing with automatic algorithm to prevent injustice and discriminations ^41^. As an example, this study showed that using the health costs as a proxy of health needs introduced a racial bias in the system, and the algorithm considered black patients healthier than equally sick white patients. This led to a reduction of 28.8% in the number of black patients to be considered with high priority of health needs ^42^. Fairness is ensured in terms of occurrence of the disease among the population that has the same characteristics represented in the dataset. Safeguards to detect and avoid biases are implemented and assessed during the training.	There are three types of bias: (1) productive bias, (2) bias that someone would qualify as unfair, and (3) discrimination bias. Algorithms cannot be unbiased. Bias in machine learning guarantees the success in modelling a certain distribution of data, according to the “No Free Lunch (NFL) Theorem”, thus solving the task of concern. The choice of cost function that the model must minimize to converge to the solution, the purpose and the use of limited training, and the test data are examples of productive bias. Also, the assumption that training data distribution will be the same of the one of the test data represents a production since in practice is often violated. However, solutions can be implemented in order to understand, mitigate or account for bias, as summarized in this survey by Ntoutsi *et al*. ^43^. The discriminatory level of ML models can be limited adding constraints to the model optimization problem. Thus, a trade-off must be found between constraints and accuracy (since it can worsen when adding to many constraints) ^44^.
**Societal and** **environmental** **wellbeing**	**Answer**	**Comments**
*Sustainable and* *environmentally friendly* *AI*	One main weakness of using AI methods is tied to the high computational demand in terms of both hardware and energy. Hence, electronic and consumables replacement/disposal are involved. Moreover, the type of model- task to be solved may require the use of especially complex and computationally expensive models ( *e.g.* text based applications). In general, more complex models require higher expense in both economical, energetic and availability terms.	A limitation of AI tools lies on the expensive hardware that is needed, especially for the training process of deep learning models. The use of GPUs is essential to increase the complexity ^45^ of the model architecture, accelerating its learning process. The more GPUs are used, the more complex the task to be solved (and hence the implemented model) can be. The price of one good GPU is around $1,800 each and computational cloud resources may also need to be rented. Moreover, specific technical methods may be adopted to reduce energy expenditure while only marginally affecting final performance ^46^.
*Social impact*	The AI system contributes to the P5 medicine purposes.	As illustrated in the previous paragraphs, the effectiveness of these AI-tools is strongly connected to the clinician-patient relationship. The empowerment of patient’s awareness on cancer diseases is aligned with the public prevention purposes to enhance current society. This has also impactful consequences on the improvement of the healthcare systems and services. Reproducibility and transparency of the scientific methods applied to develop a given AI-based system boost innovation towards the mentioned societal challenges.
*Society and democracy*	The AI system contributes to the P5 medicine purposes.	Multiple bottom-up solutions, that engage stakeholders, including vulnerable groups, may contribute to overcome societal barriers, reaching a more inclusive society. Trustworthiness of the given AI-based system constitutes a pre- condition to meet these challenges and enhance democratic values.
**Accountability**	**Answer**	**Comments**
*Auditability*	As stated in the *Fallback plan and general safety* requirement, adding redundancy to the AI method and continuous technical assessment could create a system of internal audit	Scheduling an independent audit to better test under common standards the developed technology could provide an accountable measure. The activity might be time-consuming and expensive, however the results are usually functional to align knowledge, competence, and skills useful to improve one’s approach towards innovation.
*Minimising and* *reporting negative* *impact*	In the current clinical scenario, considering the possible different levels of education and sensitivity of the end-users, other stakeholders might be involved in case of pre-identified vulnerabilities.	A survey could be implemented in the AI-tool interface in order to limit the access and use in case of specific vulnerabilities. The same solution could be adopted for minors. The tool can be used by a legal representative in case of incapacity of the end-user. In other cases, the same AI-tool interface may suggest the user to not use the tool alone.
*Documenting trade-offs*	Within the tool development, an efficient management shall ensure a continuous monitoring of technical activities and compliance ones.	A trade-off analysis is part of the R&D&I life cycle. The decision- making process provided by the AI-developer is continuously addressed to assess consequences to losing one quality, aspect or amount of something in return for gaining another quality, aspect or amount that shall in any case considered as trustworthy as the first one. Any decision shall be documented in order to be able to intervene if conditions change.
*Ability to redress*	Tests will evaluate the accuracy of the automated decision making. In case of adverse impact occurs, specific mechanisms will ensure adequate redress.	The user could be invited to self-evaluate the accuracy in terms of reproducibility of the obtained result. A survey could be implemented in the AI-tool interface to evaluate it and to report any possible adverse answer.

Table 1 summarizes those answers provided during an ALTAI session aiming at evaluating the level of trustworthiness of a given technology in order to discuss possible technical and organizational measures to be implemented in an AI-based tool to reach an acceptable level of trustworthiness. A possible limit of the self-assessment approach consists of the fact that AI controllers/developers may encounter difficulties in explaining, in plain language, how a given tool works and its consequent functionalities ^47^. This is particularly evident in the case of code interpretation, even between AI developers, but also confirmed for different domain experts. This test of the ALTAI checklist constitutes a unique exercise of semantic alignment, awareness development, and interdisciplinary training, strongly impacting on possible standardization of skills and competence involved in the AI compliance processes.

As shown in the “comments” column of the table, the checklist becomes a very useful tool for self-assessment only if it is accompanied by a series of good practices aimed at:

i) developing a common syllabus useful to align competences and skills among the different experts involved in the assessment;
ii) tailoring the core of analysis to the specific sectors where the AI-ecosystem shall be performed ( *e.g.*, health sector, workplace, mobility);
iii) addressing specific actions to firstly identify individual and group vulnerabilities, and then to overcome the relative barriers;
iv) addressing specific actions to pursue the mitigation actions beyond the development step in order to transfer to the market context the trustworthy knowledge and know-how developed during the assessment; and
v) the technical board is updated to the highest standards applicable to the given sector/market where the AI-tool is placed.

In addition, the self-assessment approach shall be promoted through policy-making incentives in order to ensure its application also without mechanisms of enforcement. In this regard, the Provisional Resolution specifies that they could constitute “ *a good starting point but cannot ensure that developers, deployers and users act fairly and guarantee the effective protection of individuals*” (Introduction, *sub* Z) and that in any cases, technology-neutral as well as specific standards shall be developed where appropriate. In particular, as far as artificial intelligence, robotics and related technologies are concerned, it suggests providing “ *mandatory compliance with legal obligations and ethical principles as laid down in the regulatory framework for AI*” to be performed through “ *an impartial, regulated and external ex-ante assessment based on concrete and defined criteria*” ( *sub* 13). Research and innovation, therefore, are those key sectors where the ALTAI checklist – or other impact assessment methodologies – could find application.

In this context, the illustrated steps will be included in every AI-related project life cycle *by design*, becoming a significant component of the research integrity and reproducibility within the scientific methodology ^48^.

## New regulatory challenges for AI in the healthcare system

In this paper, we provided an assessment of the ALTAI checklist considering the possible issues emerging while providing the evaluation for an AI-based tool applied to cancer medicine and, specifically, to early detection of melanoma skin cancer.

Firstly, the interdisciplinary approach that characterized the development of the checklist shall be applied also in the executive phase and maintained in the entre life cycle of the technology development. The methodological outcome of the analysis is needed both to interpret and then to accomplish to the technical and organizational measures to be implemented as a consequence of the risk-based analysis.

Secondly, considering that the above-mentioned Provisional Resolution identifies, as high risk ones, the applications whose “ *development, deployment and use entail a significant risk of causing injury or harm to individuals or society, in breach of fundamental rights and safety rules as laid down in Union law*” (Provisional Resolution *sub* 14), the role of AI-based systems in P5 medicine shall require the inclusion of standardized notions, tailored risks, and mechanisms to ensure either the coherence among the sectorial legislative frameworks or the opportunity to re-assess their impact any time the scientific progress could affect one of their fields of application.

Thirdly, the frontiers of P5 medicine are significantly affected by the ongoing debate on the regulatory framework for AI and Machinery Products. The opportunity to get access to health data and to re-use them for algorithms training purposes, as well as to provide further information on a given patient, is crucial for innovation in terms of effectiveness and sustainability of the developed solutions. In addition, the chance to manage health data despite the means, time, and site of their collection is a strategy that could enhance the competitiveness of the related industrial sector and, at the same time, could promote inclusiveness and awareness among citizens. In addition, according to the Machinery Products Proposal, software ensuring safety functions of machinery based on artificial intelligence are *per se* classified as “ *a high-risk machinery product due to the characteristics of artificial intelligence such as data dependency, opacity, autonomy and connectivity, which might increase very much the probability and severity of harm*”, therefore such an assessment shall be included also for those components that are assembled together with the P5 tool. This will open a series of issues related to the cost-effectiveness and machinery-accident analysis under article 5 of the mentioned Proposal.

From this perspective, the new regulatory framework on AI shall provide specific instructions on how to develop *per se* compliant hosting infrastructures and it shall address the proper consistency mechanisms to ensure that any data processing and any component of the tool could be mapped, assessed, and enabled for pre-determined purposes.

Thus, to promote a system of certification could represent a valuable solution to balance the need of procedures standardization, monitoring, and control of the level of compliance. Moreover, a multilevel system of enforcement, based on the accountability principle, and then on liability, could facilitate the establishment of a fruitful dialogue between developers and users, aiming at consolidating trust and awareness among citizens and stakeholders towards such a technological, ethical-legal revolution that AI brought in our society.

In this context, the case study provided in this paper constitutes a concrete example of the complexity of the interdisciplinary synergies that the developers and innovators shall deal with during the current transitional times determined by the regulatory, compliance, and standardization processes. It also highlights the variety of opportunities that AI-based technologies could offer to shape advanced human-centric healthcare services.

## Data availability

No data are associated with this article.

## Ethics and consent

Ethical approval and consent were not required.
