# Supplementary material for: From P4 medicine to P5 medicine: transitional times for a more human-centric approach to AI-based tools for hospitals of tomorrow
Source: Open Res Eur. 2022 Mar 9;2:33. [Version 1] doi: 10.12688/openreseurope.14524.1 (PMC10445924; doi:10.12688/openreseurope.14524.1)
Supplement: Peer review history

# Peer review: Reviewer response for version 1

**DOI:** 10.21956/openreseurope.15680.r29114

**Published:** 2022-05-19

**Authors:** Giorgia Bincoletto

**Affiliations:** 1 Faculty of Law, University of Trento, Trento, Italy

This paper provides an interdisciplinary analysis to validate an AI-based tool aiming at supporting the melanoma cancer diagnosis. The authors adopt technical, ethical, medical and legal perspectives. 

 This paper briefly reports the EU initiatives on AI in the introduction and then describes and analyses the ALTAI checklist method to assess the trustworthiness of a given AI-based technology with a particular focus in the healthcare domain. 

 This study makes a major contribution to research on gaps and strengths of the ALTAI checklist in the P5 medicine context. The table and the comments there included are very useful to compare different aspects to be taken into account. 

 Here my few comments from a legal point of view:

- As regards to the GDPR framework that has been quoted in various parts of the paper, I would suggest the authors to explicity highlight the data protection by design approach that has an important impact on the development of the AI technology and on the governace level, even in the hospital context. It might be underlined the complexity to implement both technical and organisational measures and evaluate them during the checklist assessment in the data protection phase.
- I would suggest the authors include also some (very brief) reference to the medical revice regulation that may apply to AI-based tools when used for medical purposes (e.g. diagnosis, prevention, monitoring, prediction, prognosis, treatment or alleviation of a disease); in fact, these tools shall be tested in clinical trials and eventually certified according the risks. This task is preliminary to the use of the tool. The AI Act Proposal refers to the MD Regulations explicitly as key parts of the legislative framework legislation. The authors refer to the machinery products proposal in the conclusion, but the MD assessment is important too. Even if it is not directly included in the ALTAI checklist, it is an important compliance task for hospitals.
- As regards to the comments column in the Table 1, "fundamental rights" line, I would suggest the authors include an information duty not only in the terms and conditions of the service, but also in the information notice for the medical treatment that should be detailed and very user-friendly to ensure a specific and informed consent of the patient while involved in the use of AI-based tools. 

The paper is well-written and makes and important contribution to the field.

Where applicable, are recommendations and next steps explained clearly for others to follow? (Please consider whether others in the research community would be able to implement guidelines or recommendations and/or constructively engage in the debate)

Yes

Does the article adequately reference differing views and opinions?

Yes

Are all factual statements correct, and are statements and arguments made adequately supported by citations?

Yes

Is the rationale for the Open Letter provided in sufficient detail? (Please consider whether existing challenges in the field are outlined clearly and whether the purpose of the letter is explained)

Yes

Is the Open Letter written in accessible language? (Please consider whether all subject-specific terms, concepts and abbreviations are explained)

Yes

Reviewer Expertise:

legal aspects; e-health; privacy and data protection; medical device regulation

I confirm that I have read this submission and believe that I have an appropriate level of expertise to confirm that it is of an acceptable scientific standard.

# Peer review: Reviewer response for version 1

**DOI:** 10.21956/openreseurope.15680.r29116

**Published:** 2022-05-09

**Authors:** Gabriele Tolomei

**Affiliations:** 1 Sapienza University of Rome, Rome, Italy

This paper validates the ethical-legal and technical requirements promoted by the Assessment List on Trustworthy AI (ALTAI) on an AI-based tool for supporting physicians in the early detection of melanoma skin cancer. Indeed, the healthcare domain is often considered the standard scenario when it comes to motivating the need for a trustworthy AI.

 The authors provide an in-depth assessment of the ALTAI checklist, indicating what strategies to implement for satisfying each requirement, along with their possible implications.

 Below, are my comments:

1. The analysis conducted on this work assumes the AI-based tool is trained according to the classical machine learning approach, where possibly user private training data are firstly gathered and collected by a centralized server and therein processed. However, a new, distributed learning paradigm - called federated learning (FL) has recently emerged. I would suggest the authors indicate also this approach as one of the possible strategies to mitigate data privacy issues, which may be severe, especially in the healthcare domain.
2. As far as I understand, the interdisciplinary approach proposed to validate the ALTAI checklist should be applied for the entire life cycle of the AI-based tool. I wonder, however, what the impact would be of such an effort on the feasibility of this solution.

The paper is generally well-written and easy to read, although some paragraphs would sound clearer if properly shortened. I have also spotted the following typos/errors:

- in order to do not make -> in order not to make
- since it can worsen when adding to many constraint -> since it can worsen when adding too many constraints
- a few "i.e.," and "e.g.," occurrences are boldfaced in the Table (also, a comma is missing after one of the "e.g." occurrences).

Where applicable, are recommendations and next steps explained clearly for others to follow? (Please consider whether others in the research community would be able to implement guidelines or recommendations and/or constructively engage in the debate)

Yes

Does the article adequately reference differing views and opinions?

Yes

Are all factual statements correct, and are statements and arguments made adequately supported by citations?

Yes

Is the rationale for the Open Letter provided in sufficient detail? (Please consider whether existing challenges in the field are outlined clearly and whether the purpose of the letter is explained)

Yes

Is the Open Letter written in accessible language? (Please consider whether all subject-specific terms, concepts and abbreviations are explained)

Yes

Reviewer Expertise:

explainable, robust, and collaborative machine learning

I confirm that I have read this submission and believe that I have an appropriate level of expertise to confirm that it is of an acceptable scientific standard.
